# Monocytes present age‐related changes in phospholipid concentration and decreased energy metabolism

**DOI:** 10.1111/acel.13127

**Published:** 2020-02-27

**Authors:** Mario Saare, Liina Tserel, Liis Haljasmägi, Egon Taalberg, Nadežda Peet, Margus Eimre, Rait Vetik, Külli Kingo, Kai Saks, Riin Tamm, Lili Milani, Kai Kisand, Pärt Peterson

**Affiliations:** ^1^ Molecular Pathology Research Group Institute of Biomedicine and Translational Medicine University of Tartu Tartu Estonia; ^2^ Department of Biochemistry Institute of Biomedicine and Translational Medicine University of Tartu Tartu Estonia; ^3^ Department of Pathophysiology Institute of Biomedicine and Translational Medicine University of Tartu Tartu Estonia; ^4^ Department of Dermatology and Venereology Institute of Clinical Medicine University of Tartu Tartu Estonia; ^5^ Clinic of Dermatology Tartu University Hospital Tartu Estonia; ^6^ Department of Internal Medicine Institute of Clinical Medicine University of Tartu Tartu Estonia; ^7^ Laboratory of Immune Analysis, United Laboratories Tartu University Hospital Tartu Estonia; ^8^ Estonian Genome Center Institute of Genomics University of Tartu Tartu Estonia

**Keywords:** aging, DNA methylation, glucose metabolism, monocytes, phosphatidylcholines, transcriptome

## Abstract

Age‐related changes at the cellular level include the dysregulation of metabolic and signaling pathways. Analyses of blood leukocytes have revealed a set of alterations that collectively lower their ability to fight infections and resolve inflammation later in life. We studied the transcriptomic, epigenetic, and metabolomic profiles of monocytes extracted from younger adults and individuals over the age of 65 years to map major age‐dependent changes in their cellular physiology. We found that the monocytes from older persons displayed a decrease in the expression of ribosomal and mitochondrial protein genes and exhibited hypomethylation at the HLA class I locus. Additionally, we found elevated gene expression associated with cell motility, including the *CX3CR1* and *ARID5B* genes, which have been associated with the development of atherosclerosis. Furthermore, the downregulation of two genes, *PLA2G4B* and *ALOX15B*, which belong to the arachidonic acid metabolism pathway involved in phosphatidylcholine conversion to anti‐inflammatory lipoxins, correlated with increased phosphatidylcholine content in monocytes from older individuals. We found age‐related changes in monocyte metabolic fitness, including reduced mitochondrial function and increased glycose consumption without the capacity to upregulate it during increased metabolic needs, and signs of increased oxidative stress and DNA damage. In conclusion, our results complement existing findings and elucidate the metabolic alterations that occur in monocytes during aging.

## Graphical abstract

We find age‐related changes in monocyte metabolic fitness, including increased glucose consumption without the capacity to upregulate it during increased metabolic needs, and reduced mitochondrial function together with signs of increased oxidative stress and DNA damage. Downregulation of *PLA2G4B* and *ALOX15B* expression correlates with increased phosphatidylcholine content in monocytes from older individuals and possibly lower synthesis of anti‐inflammatory lipoxins.

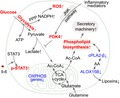

## 1 INTRODUCTION

The progression of aging is accompanied by a gradual decline of physiological and molecular processes needed to maintain the body's homeostasis (López‐Otín, Blasco, Partridge, Serrano, & Kroemer, 2013). Aging induces a well‐described set of changes in the immune system, which are collectively referred to as immunosenescence (Gruver, Hudson, & Sempowski, 2007). The weakened response to pathogenic agents and immunization is the most compelling feature of declining immune function (Giefing‐Kröll, Berger, Lepperdinger, & Grubeck‐Loebenstein, 2015). Another typical feature of aging is a state of chronic, low‐grade inflammation, also known as inflamm‐aging, which is characterized by elevated levels of proinflammatory cytokines (Franceschi et al., 2000; Sansoni et al., 2008). Considerable attention has been devoted to understanding the age‐related changes in the adaptive immune compartment, particularly in T cells (Goronzy, Hu, Kim, Jadhav, & Weyand, 2018; Johnson et al., 2017; Tserel et al., 2015; Ucar et al., 2017). However, many features of inflamm‐aging refer to a dysregulation of the innate immune system, which provides the first line of defense against invading pathogens and mediates signals to regulate the adaptive immune response. A central role in these processes has been attributed to the multifunctional monocyte cell population (Albright et al., 2016).

Human monocytes constitute approximately 10% of all peripheral blood leukocytes (Guilliams, Mildner, & Yona, 2018). The phenotyping by cell surface markers CD14 and CD16 has enabled to distinguish three main monocyte subpopulations: the classical CD14^+^CD16^−^ that represent up to 95% of monocytes, intermediate CD14^+^CD16^+^, and nonclassical CD14^−^CD16^+^ cells (Bassler, Schulte‐Schrepping, Warnat‐Herresthal, Aschenbrenner, & Schultze, 2019; Passlick, Flieger, & Ziegler‐Heitbrock, 1989) while single‐cell transcriptomics and mass cytometry has enabled even further subtyping of these immune cells (Hamers et al., 2019; Villani et al., 2017). Monocytes are important in phagocytosis, antigen presentation, inflammatory processes, and tissue repair and influence many age‐related health conditions, including atherosclerosis, inflammatory diseases, and Alzheimer's disease (Bassler et al., 2019; Jakubzick, Randolph, & Henson, 2017; Tabas & Lichtman, 2017; Wynn & Vannella, 2016; Zigmond et al., 2012). Recently, efforts have been made to elucidate the role of monocytes in aging by applying genome‐wide approaches that measure gene expression and DNA methylation (Liu, Ding, Reynolds, Lohman, & Register, 2013; Metcalf et al., 2017; Reynolds et al., 2014, 2015). These findings highlight a crucial role of monocytes in immunosenescence and suggest that aging affects the monocytic gene expression program associated with protein synthesis and cellular energy homeostasis (Metcalf et al., 2017; Reynolds et al., 2014, 2015). With age, monocytes are recruited to atherosclerotic lesions by using the CX3CR1 chemokine receptor (Tacke et al., 2007), which has higher expression in older persons (Metcalf et al., 2017). Furthermore, the transcription co‐activator ARID5B, which promotes the expression of proinflammatory markers, is upregulated in monocytes from old individuals (Liu et al., 2017). The age‐related mitochondrial dysregulation observed in many other cell types manifests itself in monocytes as a decrease in the maximal respiratory capacity (Pence & Yarbro, 2018).

Aging also influences the cytokine profile of monocytes after stimulation with TLR ligands. Specifically, studies in monocytes from elderly individuals have shown a weaker IFN‐β and IL‐1β response to influenza A virus and LPS treatment, respectively (Pillai et al., 2016; Sadeghi, Schnelle, Thoma, Nishanian, & Fahey, 1999). Additionally, Metcalf et al., (2017) reported that 5’pppRNA treatment resulted in lower induction of IFN‐α and CCL8, while LPS stimulation triggered a weaker production of IFN‐γ and IL‐1β in monocytes extracted from older donors. Moreover, low responsiveness to cytokines is characteristic of monocytes and other immune cells of aged individuals (Shen‐Orr et al., 2016).

In this study, we used genome‐wide gene expression and DNA methylation profiling of CD14^+^ monocytes extracted from individuals older than 65 years and compared the results to those found in CD14^+^ monocytes collected from healthy adults aged 23–41. We found that aging affects the expression of genes involved in protein synthesis, mitochondrial energy metabolism, and cellular motility. In addition, we found decreased expression of phospholipase A2 group 4B (*PLA2G4B*) and arachidonate 15‐lipoxygenase, type B (*ALOX15B*) mRNA, two enzymes that belong to the arachidonic acid metabolic pathway. Furthermore, we used a targeted metabolomics approach to investigate the aging‐related changes in metabolite content in monocytes and discovered increased concentrations of several phosphatidylcholine (PC) species. Similarly, we found that the inhibition of phospholipase A2 activity in a monocytic cell line results in elevated levels of PCs. Based on these large‐scale screens, we performed a comprehensive analysis of key markers of cellular physiology to compare the steady‐state and LPS‐stimulated monocytes extracted from old and young individuals and discovered important age‐related disturbances in energy metabolism.

## 2 RESULTS

### 2.1 Monocytes from older individuals display gene expression hallmarks of aging and reveal an impact on arachidonic acid metabolism

**Figure 1:**
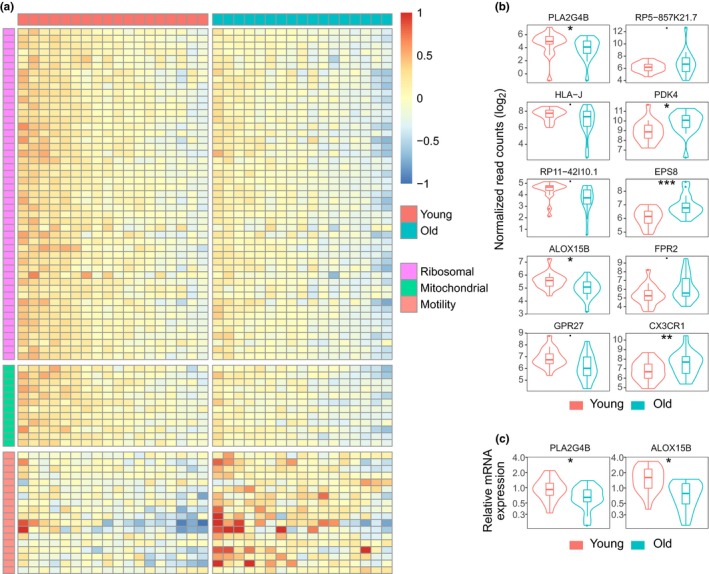
Expression analysis of monocytes extracted from young adults and old individuals. (a) Heatmap of age‐related differentially expressed genes that encode ribosomal or mitochondrial proteins or affect cell motility. (b) Gene expression comparison of top 5 downregulated (first column) and top 5 upregulated (second column) genes in monocytes between age groups. (c) The relative expression of genes *PLA2G4B* and *ALOX15B* in monocytes extracted from young adults (20 samples) and old persons (21 samples). Monocytes were isolated from fresh samples and the cell pellets stored frozen until RNA extraction. Asterisks show FDR‐adjusted *p*‐value ranges: *p* < .1; *p < .05; **p < .01; ***p < .001 (Wald test in (b) and ANOVA in (c))

We performed genome‐wide mRNA expression profiling to identify the genes that are differentially expressed in CD14^+^ monocytes from younger adults (average age 35.7 years, standard deviation 4.9) and older individuals (average age 71.6 years, standard deviation 3.1) (Table S1). We found that a large fraction of genes encoding ribosomal proteins (~50 out of 80) had a moderate decline of approximately 10%–20% in the CD14^+^ cells extracted from the older subjects (Figure 1a and Table S2). The second largest group of genes that displayed reduced expression in monocytes of older individuals was associated with mitochondrial functions, including oxidative phosphorylation and transport through the mitochondrial membranes (Figure 1a and Table S2 & S3). We found that the largest group of genes, the expression of which was increased with age, was associated with cell motility and migration (Figure 1a and Table S2 & S3). The three out of five top upregulated genes—epidermal growth factor receptor pathway substrate 8 (*EPS8*), formyl peptide receptor 1 (*FPR2*), and C‐X3‐C motif chemokine receptor 1 (*CX3CR1*)—were associated with cell movement, chemotaxis, and inflammatory processes (Aoki et al., 2016; Imai et al., 1997; Tiffany et al., 2001) (Figure 1b). In particular, CX3CR1, which binds fractalkine thereby promoting monocyte migration and cell adhesion, has been shown to direct monocytes to atherosclerotic lesions in blood vessels (Tacke et al., 2007). Our list of upregulated genes also includes the transcription factor AT‐rich interaction domain 5B (ARID5B), which has been shown to activate adipogenic gene expression and promote the expression on atherogenic markers in monocytes (Liu et al., 2017; Whitson, Tsark, Huang, & Itakura, 2003; Yamakawa, Whitson, Li, & Itakura, 2008) (Table S2). Another highly upregulated gene was pyruvate dehydrogenase kinase 4 (*PDK4*), which inhibits the pyruvate dehydrogenase complex, thereby shifting glucose metabolism from oxidative phosphorylation to lactate production (Park & Jeoung, 2016) (Figure 1b).

Interestingly, two of the top downregulated genes—phospholipase A2 group IV family (*PLA2G4B*) and arachidonate 15‐lipoxygenase type B (*ALOX15B*)—encode enzymes that belong to the arachidonic acid metabolism pathway (KEGG: ko00590) (Figure 1b,c). The *PLA2G4B* encoded cPLA_2_‐β enzyme converts phosphatidylcholines (PCs) to arachidonic acid, which is a known precursor of many important pro‐ and anti‐inflammatory mediators. The ALOX15B protein can catalyze the formation of 15(S)‐ and 8(S)‐hydroxyeicosatetraenoic (HETE) acids, which are able to trigger PPAR‐γ‐mediating anti‐inflammatory responses, or can be metabolized further to lipoxins that are endogenous anti‐inflammatory and pro‐resolving molecules counteracting chronic inflammation (Chandrasekharan & Sharma‐Walia, 2015; Chawla, 2010; Ivanova et al., 2015). Other top downregulated genes included the major histocompatibility complex class I pseudogene J (*HLA‐J*), the uncharacterized long noncoding RNA gene RP11‐42I10.1, and the G protein‐coupled receptor 27 (*GPR27*) (Figure 1b). Thus, our analysis detected major groups of genes that are associated with the hallmarks of aging and highlighted the age‐related downregulation of two enzymes in the arachidonic acid metabolism pathway, suggesting impaired regulation of chronic inflammation.

### 2.2 DNA methylation changes in monocytes of old subjects occur at known aging marker sites

**Figure 2:**
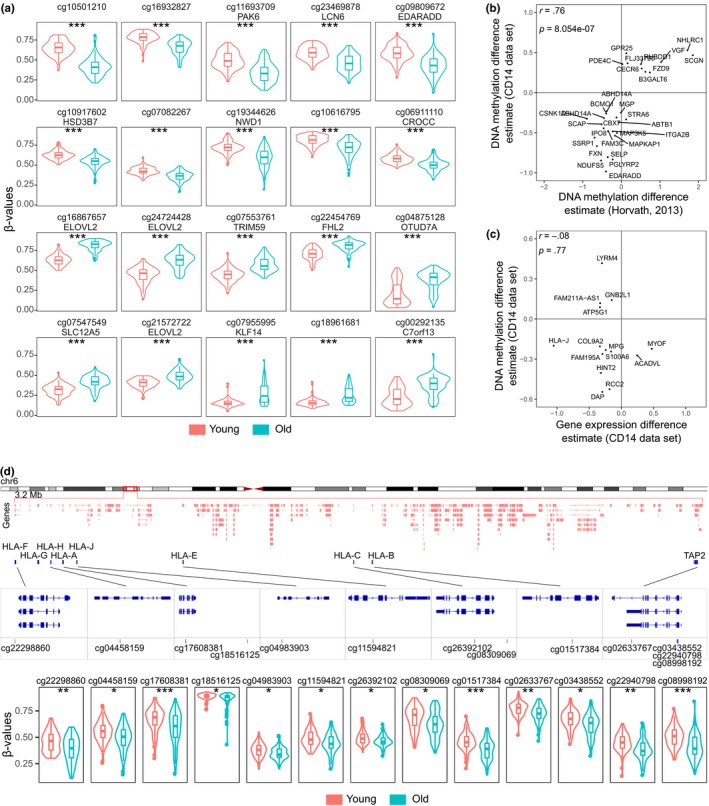
DNA methylation analysis of monocytes extracted from young and old individuals. (a) Comparison of top 10 hypomethylated (first two rows) and top 10 hypermethylated (last two rows) CpG positions in monocytes between age groups. (b) Correlation between DMPs and epigenetic clock CpGs described in Horvath, 2013. The labels show the nearest genes. (c) Correlation between DMPs and nearest differentially expressed genes. (d) The 3.2 Mb MHC I region depicting genes and DMPs. The lower panel shows the comparison of DNA methylation level of individual CpG sites in monocytes between age groups (sample sizes: 93 young and 91 old individuals). Monocytes were isolated from fresh samples and the cell pellets frozen until DNA extraction. Asterisks show FDR‐adjusted *p*‐value ranges: *p < .05; **p < .01; ***p < .001 (moderated *t* test in (a) and (d)). The *r* in (b) and (c) shows the Pearson's correlation coefficient

We analyzed genome‐wide DNA methylation patterns with the Illumina Infinium 450 K BeadChip technology and compared samples from young adults (average age 34.2 years, standard deviation 4.7) to older individuals (average age 71.7 years, standard deviation 3.3). Altogether, we detected 2,967 CpG sites that displayed differential methylation between age groups (Table S4). We found that the top hypermethylated sites in samples from old individuals include CpGs at *ELOVL2* and *FHL2* gene loci, which have been shown to strongly correlate with age (Garagnani et al., 2012) (Figure 2a). Additionally, the direction and the magnitude of change in a set of 30 CpG sites correlated strongly with the age‐predicting set of CpGs that are part of the epigenetic clock (Horvath, 2013) (Figure 2b). Interestingly, only a small number of the differentially expressed genes had differentially methylated CpG sites (Figure 2c). These sites were located mainly in gene bodies, and accordingly, the gene expression and the associated DMPs did not correlate; rather, both were decreased in monocytes from older individuals.

We detected a consistent hypomethylation of CpG sites at MHC class I loci in samples from older individuals (Figure 2d). The majority of the DMPs within the HLA region were located within gene bodies, including the hypomethylated cg04983903 site within the first intron of the *HLA‐J* pseudogene, which was the only MHC I locus that displayed age‐related differential expression (Figure 1b). In addition, several sites were hypomethylated within the antigen peptide transporter 2 (TAP2) gene region.

### 2.3 Age affects phosphatidylcholine content in monocytes

**Figure 3:**
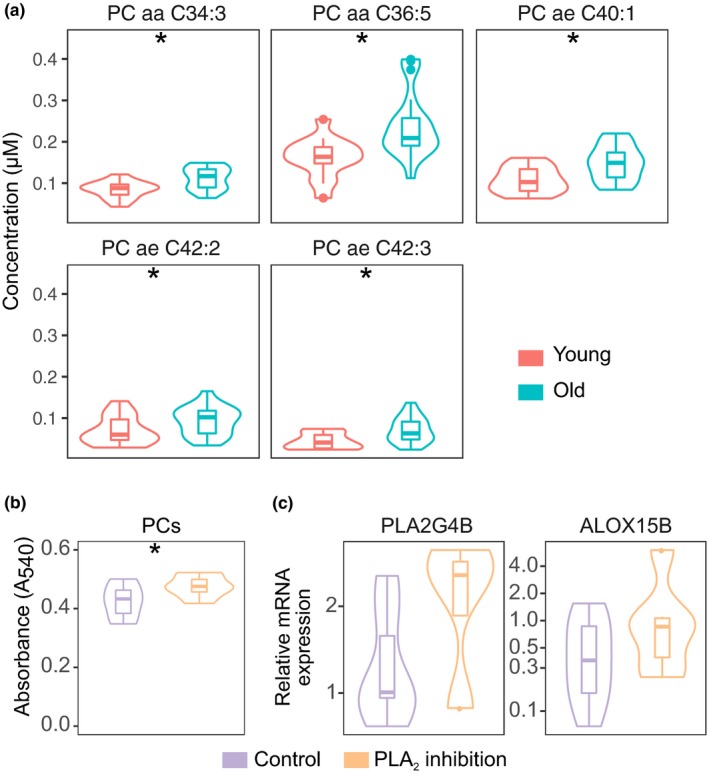
Aging affects phosphatidylcholine content of monocytes. (a) Phosphatidylcholine species that display age‐related differences in their concentration. (b) Difference in phosphatidylcholine content in THP‐1 cells after phospholipase A2 inhibition with PACOCF_3_. One‐tailed *t* test: *p < .05. (c) The relative expression of genes *PLA2G4B* and *ALOX15B* in THP‐1 cells after phospholipase A2 inhibition with PACOCF_3_. The sample size was 14 young and 19 old individuals in (a) and 7 independent cell cultures in (b,c). Monocytes were isolated from fresh samples and stored frozen until the lipid content was analyzed. Asterisks show the FDR‐adjusted *p*‐value range *p < .05 (ANOVA)

The downregulation of the *PLA2G4B* gene encoding cPLA_2_‐β raised the possibility that PCs which serve as a major input of the arachidonic acid metabolism pathway could have altered concentrations in the monocytes of old individuals. To test this hypothesis, we performed a targeted screening of 188 metabolites with the Biocrates AbsoluteIDQ p180 kit comparing monocyte cell extracts from young adults (average age 33.1 years, standard deviation 3.1) to samples from old individuals (average age 78.3 years, standard deviation 7.7). As lipid‐lowering medication may affect phosphatidylcholine levels, we excluded individuals who had statin treatment from our analysis (the treatment decreased the number and statistical significance of age‐affected phosphatidylcholine (PC) species, Figure S1a). Overall, we were able to reliably detect 77 metabolites, including 56 PCs (Table S6). The statistical comparison of the age groups provided evidence that five PC species, collectively representing 41 potential isomeric molecules, had a significantly higher concentration in cells extracted from older individuals (Figure 3a).

To determine whether the concentration of PCs depends on the availability of phospholipase A2 activity, we inhibited PLA_2_ enzymes with the small‐molecule inhibitor PACOCF3 and measured the overall PC levels in the monocytic cell line THP‐1. We found that the inhibition of PLA_2_ enzymes significantly increased the concentration of PCs in the THP‐1 cells (Figure 3b). We also noted that the mRNA levels of *PLA2G4B* or *ALOX15B* genes in THP‐1 cells did not significantly change after PLA_2_ inhibition (Figure 3c). We conclude that the PC concentrations are affected by the availability of phospholipase A2 activity in monocytes.

### 2.4 Increased glucose uptake and oxidative stress are age‐dependent

**Figure 4:**
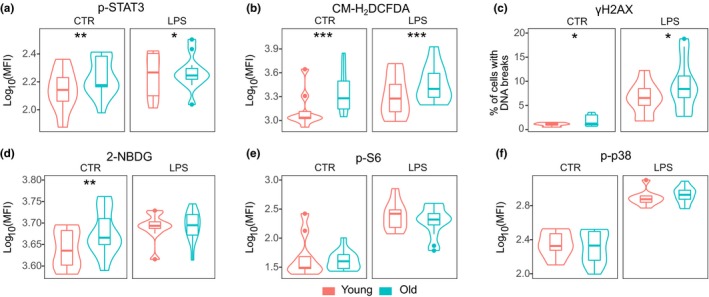
Screening of markers of cellular physiology and stress. Age‐related differences of mock‐ and LPS‐treated monocytes in (a) the inflammation marker p‐STAT3, (b) oxidative stress indicator CM‐H_2_DCFDA, (c) the double‐stranded DNA break marker γH2AX, (d) the glucose uptake indicator 2‐NBDG, (e) the mTOR pathway activity marker p‐S6, and (f) the general cellular stress marker p‐p38. Asterisks show the *p*‐value ranges: *p < .05; **p < .01; ***p < .001 (ANOVA)

The differentially expressed genes in young and old CD14^+^ monocytes prompted us to investigate more deeply the cellular functions related to these gene sets. We used fluorescence emitting substrates and labeled antibodies against key markers of cellular stress and metabolic state that are known to be associated with inflammatory processes, such as phosphorylated STAT3, phosphorylated histone H2AX (γH2AX), phosphorylated p38, and phosphorylated ribosomal protein S6 (Arthur & Ley, 2013; Bromberg & Darnell, 2000; Jastrzebski, Hannan, Tchoubrieva, Hannan, & Pearson, 2007; Rogakou, Pilch, Orr, Ivanova, & Bonner, 1998). Additionally, we used the glucose analog 2‐NBDG and CM‐H_2_DCFDA to estimate the uptake of glucose and the presence of ROS in monocytes, respectively (Eruslanov & Kusmartsev, 2010; Yamada, Saito, Matsuoka, & Inagaki, 2007). Experiments with steady‐state monocytes were complemented with LPS‐treated cells to monitor the age‐related responses to the immunological challenge. The flow cytometric analyses were performed with monocytes extracted from young adults (average age 29.6 years, standard deviation 3.7) and from old individuals (average age 79.4 years, standard deviation 6.3). All analyses excluded older individuals who were using lipid‐lowering medication, although the treatment did not affect the outcome (compare Figures 4 and S1b–g).

In agreement with the overall low‐grade inflammation and published research (Shen‐Orr et al., 2016), the monocytes sorted from older individuals showed significantly higher baseline levels of the inflammation marker p‐STAT3 than those of younger individuals (Figure 4a). STAT3 is phosphorylated downstream of several cytokines including IL‐6 that is one of the mediators upregulated during inflamm‐aging (Wikby et al., 2006). LPS treatment was able to augment the p‐STAT3 signal in cells from both age groups albeit with slightly lower dynamics in aged individuals (Table S7). Aging at the cellular level also features a higher concentration of ROS within cells, which has been associated with DNA damage (Davalli, Mitic, Caporali, Lauriola, & D'Arca, 2016). On average, we found twofold higher ROS production in unstimulated monocytes in old versus young individuals (Figure 4b). Furthermore, the percentage of monocytes displaying the histone γH2AX signal, a marker of DNA double‐stranded breaks, was increased about 1.5 times among older subjects (Figure 4c). Finally, LPS treatment resulted in efficient oxidative burst and highly elevated levels of γH2AX signals in the monocytes of both younger and older persons (Table S7).

Interestingly, we found that monocytes from older individuals were more ready to take up the glucose analog 2‐NBDG (Figure 4d). Moreover, treatment with LPS did not affect the uptake in cells from older donors, whereas monocytes from young individuals reacted to LPS by significantly increasing the 2‐NBDG uptake (Table S7). The tendency to take up more 2‐NBDG was evident both in classical (CD16^−^) and nonclassical (CD16^+^) monocytes, although the small sample size limited the statistical evaluation (Figure S2a). To further assess the metabolic state of monocytes in the different age groups, we also tested the activation of the mammalian target of rapamycin (mTOR) signaling cascade, which converges many metabolic and other cellular signals and promotes anabolic processes, thereby leading to cell proliferation and growth (Loewith & Hall, 2011; Wullschleger, Loewith, & Hall, 2006). We measured the phosphorylation of the ribosomal S6 protein, which is the target of ribosomal protein S6 kinase beta‐1 (p70‐S6K1), a downstream target of the mTOR pathway (Magnuson, Ekim, & Fingar, 2012). We did not find significant differences in the level of S6 phosphorylation between the age groups with or without LPS treatment (Figure 4e). However, LPS treatment alone resulted in a dramatic 8‐ to 10‐fold increase of the phosphorylated S6 (pS6) in both age groups indicating that the cells were equally responsive to external stimuli (Table S7). However, the subtle in vivo differences in pS6 at the cell population level are not easily detectable, as the nutrients in the culture medium tend to activate mTOR (Sengupta, Peterson, & Sabatini, 2010).

We also tested whether monocytes from older subjects display general cellular stress response by measuring the phosphorylated form of p38 mitogen‐activated protein kinase (MAPK), which is activated by cytokines and itself regulates the expression of many inflammatory mediators (Arthur & Ley, 2013). Interestingly, our results show that the phosphorylation of p38 in monocytes is not age‐dependent (Figure 4f); however, the stress marker was strongly activated when the cells were treated with LPS irrespective of age (Table [Link], [Link]).

### 2.5 Monocytes from older subjects contain more mitochondria albeit with lower functional capacity

**Figure 5:**
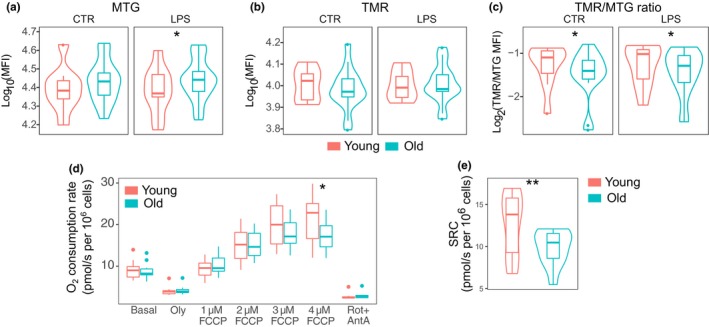
The decline of cellular respiration of monocytes during aging. Age‐related differences of mock‐ and LPS‐treated monocytes in (a) the overall amount of mitochondria by MitoTracker Green (MTG) staining, (b) the amount of functional mitochondria by tetramethylrhodamine (TMR) staining, and (c) the ratio of functional to total mitochondria. (d) Analysis of the cellular respiration. Oly—Oligomycin; FCCP—p‐trifluoromethoxyphenylhydrazone; Rot + AntA—Rotenone and Antimycin A; SRC—spare respiratory capacity. (e) Comparison of spare respiratory capacity of monocytes between age groups. The sample sizes were 9 young and 16 old individuals for the experiments in (a–c) and 9 young and 9 old individuals in (d,e). The experiments were performed with fresh cells. Asterisks show the *p*‐value ranges: *p < .05; **p < .01; ***p < .001 (ANOVA)

Published data have shown that the cellular respiratory capacity steadily declines with age, which is a consequence of dysfunctional mitochondria (Bratic & Larsson, 2013). The higher concentration of ROS in monocytes from old individuals (Figure 4b) (Jacinto et al., 2018), as well as the downregulated expression of OXPHOS‐related genes (Figure 1a), prompted us to test the properties of mitochondria in younger and older subjects. We measured the total mitochondrial mass by the fluorescence signal of MTG and estimated the mitochondrial membrane potential by analyzing the mitochondrial uptake of the membrane potential‐sensitive dye TMR. We noticed a trend for increased mitochondrial mass but lower mitochondrial membrane potential in monocytes from aged persons in both tested conditions (Figure 5a,b and Table [Link], [Link]). Importantly, the cell‐wise ratios of the dyes revealed significantly lower membrane potential per mitochondrion and thus impaired mitochondrial health in aged monocytes (Figure 5c). Interestingly, the mitochondrial mass differences were clearly detected in the nonclassical (CD16^+^) monocytes (Figure S2b, Table [Link], [Link]). However, the mitochondrial membrane potential and its ratio to mitochondrial mass were decreased in both cell types of old individuals (Figure S2c–d, Table [Link], [Link]).

To complement the findings on mitochondrial properties, we assayed the oxygen consumption rate (OCR) of monocytes extracted from both younger (average age 29.6 years, standard deviation 3.7) and older individuals (average age 82.3 years, standard deviation 4.7). We determined the basal and maximal respiration of the cells by chemically inhibiting or modifying the different electron transfer complexes in the mitochondrial membrane (Figure 5d). Based on the measurements, we calculated the spare respiratory capacity (SRC) by subtracting the basal OCR from the maximal OCR and found a significantly lower SRC in cells from older individuals (Figure 5e).

## 3 DISCUSSION

In this study, we compared the genome‐wide gene expression and DNA methylation patterns of CD14^+^ monocytes extracted from young and old individuals and complemented these findings with thorough investigations on age‐related changes of metabolite concentrations and key markers of cellular physiology. Although we concentrated our study on the classical CD14^+^ monocyte subpopulation that is most abundant in the circulation, in parallel we derived some additional hints that CD16^+^ monocytes may undergo similar age‐related changes. The differences in mRNA expression lie mainly in genes with role in protein synthesis and mitochondrial respiration cascade and are in line with previous data (Kumar et al., 2013; Peters et al., 2015; Reynolds et al., 2015; van den Akker et al., 2014; Zahn et al., 2006).

The negative effects of aging on protein synthesis and energy metabolism are major hallmarks of aging (López‐Otín et al., 2013). The aging process in many organisms is associated with alterations of the ribosome biogenesis, an overall decline of protein synthesis and the age‐related reduction of rRNA expression (D'Aquila et al., 2017), suggesting that both RNA polymerase II‐ and III‐dependent transcription are affected. The ribosomal protein and RNA expression are controlled by the mTOR pathway (Iadevaia, Liu, & Proud, 2014), although we could not demonstrate the decreased mTOR activity in aged monocytes. The downregulation of electron transport chain components also correlates with age‐related diseases and shorter lifespan (Bratic & Larsson, 2013). In agreement, we detected lower mitochondrial membrane potential and higher levels of ROS in monocytes from old individuals. ROS could be derived from damaged mitochondria, or due to increased activity of NADPH oxidase caused by inflammatory signals. However, long‐term exposure to ROS can also lead to mitohormesis, which serves as a protective mechanism against mitochondrial oxidants (Yun & Finkel, 2014). Thus, the elevated level of ROS in monocytes extracted from old subjects may not serve only as a source of cellular stress, but also counteract it. Interestingly, although we found a tendency for increased phospho‐p38 and pS6 in older individuals, in our study these differences did not reach significance level though they have been shown to increase in stress, aging, and senescence (Arthur & Ley, 2013; Callender et al., 2018; Vukmanovic‐Stejic et al., 2018).

Of the specific top‐listed differentially expressed genes, two upregulated genes, *CX3CR1* and *ARID5B*, have been previously associated with atherosclerosis (Landsman et al., 2009; Liu et al., 2017). The chemokine receptor CX3CR1 promotes the migration of monocytes and macrophages to sites of atherosclerotic lesions and its expression correlates with age (Landsman et al., 2009; Metcalf et al., 2017). ARID5B is a transcription factor known to promote the activation of lipogenesis‐related genes and to affect cell motility (Yamakawa et al., 2008). Furthermore, Liu et al. (2017) describe a strong link between the knock‐down of *ARID5B* mRNA and the decreased expression of atherosclerosis‐promoting factors. Our analysis lends support to the earlier findings and strengthens the notion that these two proteins have atherogenic properties, as age is one of the major risk factors for atherosclerosis.

We also show that age‐related changes in DNA methylation occur at CpG sites that are now considered markers of aging irrespective of the analyzed tissue (Horvath, 2013). Many of the sites are part of the so‐called epigenetic clock that correlates with the chronological age of a person, but additionally could predict health issues if the correlation is altered (Horvath, 2013). However, the relationship between the DNA methylation and gene expression is complex and most of the DNA methylation changes do not result in a significant impact on gene expression (van Eijk et al., 2012; Horvath, 2013). Unsurprisingly, our study, which used relatively small sample sizes for the analyses, found only a weak correlation between the differentially expressed genes and DNA methylation sites. Reynolds et al., 2014 have shown that a larger sample size permits the detection of subtle correlations between the data sets, but the functional consequences remain to be elucidated.

Interestingly, we found that *PDK4* mRNA was strongly upregulated in CD14^+^ monocytes from old individuals. Inflammatory signals, such as LPS, increase the expression of PDK4 via JNK pathway (Park & Jeoung, 2016). In turn, increased PDK4 expression inhibits the pyruvate dehydrogenase complex by phosphorylation of its E1α component (PDHE1α), which shifts glucose metabolism from oxidative phosphorylation toward lactate production. To meet enhanced energy demands, many immune cell types upregulate glycolytic ATP production during their activation (O'Neill, Kishton, & Rathmell, 2016). Our data suggest similar glycolytic upregulation during inflamm‐aging as the monocytes from older donors displayed higher uptake of the glucose analog 2‐NBDG. It is noteworthy that LPS stimulation enhanced the 2‐NBDG uptake only in monocytes extracted from younger adults. This suggests that the low‐grade inflammation, which is experienced by monocytes from older people, has already set the glucose utilization speed to its upper limit. The decreased expression of genes related to OXPHOS, decreased mitochondrial potential, and reduced SRC indicate that the balance of energy metabolism has tipped with age from OXPHOS toward aerobic glycolysis. This type of metabolic rewiring has been associated with the generation of “trained innate immunity” that potentiates innate responses against pathogens but is possibly also able to aggravate inflammatory pathology (Bekkering et al., 2016; Cheng et al., 2014). Monocytes from aged persons have some similarities to “trained” innate cells but also some important differences. The switch to aerobic glycolysis in healthy monocytes and macrophages helps to obtain fast energy supply and to generate metabolites to carry out the immediate protective function of the cells: cytokine production, oxidative burst for microbicidal function, phagocytosis, and antigen presentation for adaptive immunity (O'Neill et al., 2016). Data from published and current research indicate that these functions are impaired in monocytes from aged persons that can result from the cellular energy deficit (Albright et al., 2016; Metcalf et al., 2017; Pence & Yarbro, 2018; Shen‐Orr et al., 2016). The metabolic fitness of monocytes from aged persons seems to be impaired with decreased mitochondrial respiratory reserve and restricted capacity to utilize additional glucose. As a result, the limited energy supply cannot be spared for ribosomal biogenesis, which is one of the most energy‐consuming processes in the cell (MacInnes, 2016), and for cytokine production, which is also impaired in aged monocytes according to many previous studies (Metcalf et al., 2017; Pillai et al., 2016; Sadeghi et al., 1999).

The top list of downregulated genes featured *PLA2G4B* and *ALOX15B*, which encode enzymes that belong to consecutive steps in the arachidonic acid metabolism pathway. Because *PLA2G4B* encoded cPLA2‐β utilizes PCs, we analyzed the concentration of a panel of metabolites to discover age‐related differences that could be due to the limited availability of the enzyme. Older individuals had a higher concentration of several PC species in monocytes, and experiments with the monocytic THP‐1 cell line further showed that inhibiting the phospholipase A2 activity increased the amount of PCs content in the cells. Although it is not currently possible to decipher the connection between the specific phospholipases and PC species, we believe that our results establish a basis to investigate the age‐dependent changes in phospholipid turnover in monocytes. Moreover, PC content increases during monocyte activation as the secretory machinery expands to meet the cellular needs for increased cytokine secretion (Langston, Shibata, & Horng, 2017), which in aging may represent the senescence‐associated secretory phenotype (Shen‐Orr et al., 2016). Furthermore, many downstream products of the arachidonic acid metabolism pathway are important inflammatory signaling molecules, such as prostaglandins, thromboxanes, leukotrienes, and hydroxyeicosatetraenoic acids, and it would be important to determine their exact role in the context of aging. Our results indicate that the balance of pro‐ and anti‐inflammatory mediators of the pathway may favor inflammation in aged individuals. Additionally, cell type‐specific analysis of metabolite content would be helpful in determining how the different subsets affect the repertoire of blood plasma metabolites that have been established previously (Yu et al., 2012).

In summary, we show that CD14^+^ monocytes from older individuals undergo changes in gene expression and DNA methylation changes that reflect the hallmarks of aging. These results are complemented by new insights into changes in phospholipids, and the metabolic and signaling status of monocytes during aging. Considering the central role of monocytes bridging the signals of innate and adaptive immunity, it would be highly valuable to advance our understanding of the metabolic processes that occur in monocytes and to use it to mitigate age‐related conditions.

## 4 EXPERIMENTAL PROCEDURES

All experimental procedures are described in detail in the Supporting Information file.

## CONFLICT OF INTEREST

The authors declare no conflict of interest.

## AUTHOR CONTRIBUTIONS

M.S. designed and analyzed the qPCR, metabolomics, and phospholipase inhibition experiments, analyzed the genome‐wide transcriptomic, DNA methylation, flow cytometric, and cell respiration data sets; L.T. and L.M. designed and performed the genome‐wide transcriptomic and DNA methylation experiments; K.Kisand and L.H. designed, performed, and analyzed the flow cytometry experiments; N.P. and M.E. designed and performed the cell respiration experiments; E.T. performed and analyzed the metabolomics experiment; R.V. performed the phospholipase inhibition assay; K.Kingo, K.S. and R.T. are responsible for the collection and documentation of blood donor samples; L.M., K.Kisand and P.P. supervised the overall project; M.S., K.Kisand and P.P. wrote the manuscript.

## Supporting information

FigureS1‐S6

TableS1

TableS2

TableS3

TableS4

TableS5

TableS6

TableS7

TableS8

Supinfo

## References

[acel13127-bib-0001] Albright, J. M. , Dunn, R. C. , Shults, J. A. , Boe, D. M. , Afshar, M. , & Kovacs, E. J. (2016). Advanced age alters monocyte and macrophage responses. Antioxidants & Redox Signaling, 25(15), 805–815. 10.1089/ars.2016.6691 27357201 PMC5107740

[acel13127-bib-0002] Aoki, K. , Maeda, F. , Nagasako, T. , Mochizuki, Y. , Uchida, S. , & Ikenouchi, J. (2016). A RhoA and Rnd3 cycle regulates actin reassembly during membrane blebbing. Proceedings of the National Academy of Sciences of the United States of America, 113(13), E1863–1871. 10.1073/pnas.1600968113 26976596 PMC4822640

[acel13127-bib-0003] Arthur, J. S. , & Ley, S. C. (2013). Mitogen‐activated protein kinases in innate immunity. Nature Reviews Immunology, 13(9), 679–692. 10.1038/nri3495 23954936

[acel13127-bib-0004] Bassler, K. , Schulte‐Schrepping, J. , Warnat‐Herresthal, S. , Aschenbrenner, A. C. , & Schultze, J. L. (2019). The Myeloid Cell Compartment‐Cell by Cell. Annual Review of Immunology, 37(1), 269–293. 10.1146/annurev-immunol-042718-041728 30649988

[acel13127-bib-0005] Bekkering, S. , van den Munckhof, I. , Nielen, T. , Lamfers, E. , Dinarello, C. , Rutten, J. , … Riksen, N. P. (2016). Innate immune cell activation and epigenetic remodeling in symptomatic and asymptomatic atherosclerosis in humans in vivo. Atherosclerosis, 254, 228–236. 10.1016/j.atherosclerosis.2016.10.019 27764724

[acel13127-bib-0006] Bratic, A. , & Larsson, N. G. (2013). The role of mitochondria in aging. Journal of Clinical Investigation, 123(3), 951–957. 10.1172/JCI64125 23454757 PMC3582127

[acel13127-bib-0007] Bromberg, J. , & Darnell, J. E. (2000). The role of STATs in transcriptional control and their impact on cellular function. Oncogene, 19(21), 2468–2473. 10.1038/sj.onc.1203476 10851045

[acel13127-bib-0008] Callender, L. A. , Carroll, E. C. , Beal, R. W. J. , Chambers, E. S. , Nourshargh, S. , Akbar, A. N. , & Henson, S. M. (2018). Human CD8 + EMRA T cells display a senescence‐associated secretory phenotype regulated by p38 MAPK. Aging Cell, 17(1), e12675 10.1111/acel.12675 PMC5770853 29024417

[acel13127-bib-0009] Chandrasekharan, J. A. , & Sharma‐Walia, N. (2015). Lipoxins: Nature's way to resolve inflammation. Journal of Inflammation Research, 8, 181–192. 10.2147/JIR.S90380 26457057 PMC4598198

[acel13127-bib-0010] Chawla, A. (2010). Control of macrophage activation and function by PPARs. Circulation Research, 106(10), 1559–1569. 10.1161/CIRCRESAHA.110.216523 20508200 PMC2897247

[acel13127-bib-0011] Cheng, S. C. , Quintin, J. , Cramer, R. A. , Shepardson, K. M. , Saeed, S. , Kumar, V. , … Netea, M. G. (2014). mTOR‐ and HIF‐1α‐mediated aerobic glycolysis as metabolic basis for trained immunity. Science, 345(6204), 1250684 10.1126/science.1250684 25258083 PMC4226238

[acel13127-bib-0012] D'Aquila, P. , Montesanto, A. , Mandalà, M. , Garasto, S. , Mari, V. , Corsonello, A. , … Passarino, G. (2017). Methylation of the ribosomal RNA gene promoter is associated with aging and age‐related decline. Aging Cell, 16(5), 966–975. 10.1111/acel.12603 28625020 PMC5595699

[acel13127-bib-0013] Davalli, P. , Mitic, T. , Caporali, A. , Lauriola, A. , & D'Arca, D. (2016). ROS, cell senescence, and novel molecular mechanisms in aging and age‐related diseases. Oxidative Medicine and Cellular Longevity, 2016, 3565127 10.1155/2016/3565127 27247702 PMC4877482

[acel13127-bib-0014] Eruslanov, E. , & Kusmartsev, S. (2010). Identification of ROS using oxidized DCFDA and flow‐cytometry. Methods in Molecular Biology, 594, 57–72. 10.1007/978-1-60761-411-1_4 20072909

[acel13127-bib-0015] Franceschi, C. , Bonafè, M. , Valensin, S. , Olivieri, F. , De Luca, M. , Ottaviani, E. , & De Benedictis, G. (2000). Inflamm‐aging: An Evolutionary Perspective on Immunosenescence. Annals of the New York Academy of Sciences, 908(1), 244–254. 10.1111/j.1749-6632.2000.tb06651.x 10911963

[acel13127-bib-0016] Garagnani, P. , Bacalini, M. G. , Pirazzini, C. , Gori, D. , Giuliani, C. , & Mari, D. (2012). Methylation of ELOVL2 gene as a new epigenetic marker of age. Aging Cell, 11(6), 1132–1134. 10.1111/acel.12005 23061750

[acel13127-bib-0017] Giefing‐Kröll, C. , Berger, P. , Lepperdinger, G. , & Grubeck‐Loebenstein, B. (2015). How sex and age affect immune responses, susceptibility to infections, and response to vaccination. Aging Cell, 14(3), 309–321. 10.1111/acel.12326 25720438 PMC4406660

[acel13127-bib-0018] Goronzy, J. J. , Hu, B. , Kim, C. , Jadhav, R. R. , & Weyand, C. M. (2018). Epigenetics of T cell aging. Journal of Leukocyte Biology, 104(4), 691–699. 10.1002/JLB.1RI0418-160R 29947427 PMC6162101

[acel13127-bib-0019] Gruver, A. L. , Hudson, L. L. , & Sempowski, G. D. (2007). Immunosenescence of ageing. The Journal of Pathology, 211(2), 144–156. 10.1002/path.2104 17200946 PMC1931833

[acel13127-bib-0020] Guilliams, M. , Mildner, A. , & Yona, S. (2018). Developmental and functional heterogeneity of monocytes. Immunity, 49(4), 595–613. 10.1016/j.immuni.2018.10.005 30332628

[acel13127-bib-0021] Hamers, A. A. J. , Dinh, H. Q. , Thomas, G. D. , Marcovecchio, P. , Blatchley, A. , Nakao, C. S. , … Hedrick, C. C. (2019). Human monocyte heterogeneity as revealed by high‐dimensional mass cytometry. Arteriosclerosis, Thrombosis, and Vascular Biology, 39(1), 25–36. 10.1161/ATVBAHA.118.311022 PMC6697379 30580568

[acel13127-bib-0022] Horvath, S. (2013). DNA methylation age of human tissues and cell types. Genome Biology, 14(10), R115 10.1186/gb-2013-14-10-r115 24138928 PMC4015143

[acel13127-bib-0023] Iadevaia, V. , Liu, R. , & Proud, C. G. (2014). mTORC1 signaling controls multiple steps in ribosome biogenesis. Seminars in Cell & Developmental Biology, 36, 113–120. 10.1016/j.semcdb.2014.08.004 25148809

[acel13127-bib-0024] Imai, T. , Hieshima, K. , Haskell, C. , Baba, M. , Nagira, M. , Nishimura, M. , … Yoshie, O. (1997). Identification and molecular characterization of fractalkine receptor CX3CR1, which mediates both leukocyte migration and adhesion. Cell, 91(4), 521–530. 10.1016/S0092-8674(00)80438-9 9390561

[acel13127-bib-0025] Ivanova, E. A. , Parolari, A. , Myasoedova, V. , Melnichenko, A. A. , Bobryshev, Y. V. , & Orekhov, A. N. (2015). Peroxisome proliferator‐activated receptor (PPAR) gamma in cardiovascular disorders and cardiovascular surgery. Journal of Cardiology, 66(4), 271–278. 10.1016/j.jjcc.2015.05.004 26072262

[acel13127-bib-0026] Jacinto, T. A. , Meireles, G. S. , Dias, A. T. , Aires, R. , Porto, M. L. , Gava, A. L. , … Meyrelles, S. S. (2018). Increased ROS production and DNA damage in monocytes are biomarkers of aging and atherosclerosis. Biological Research, 51(1), 33 10.1186/s40659-018-0182-7 30185234 PMC6123971

[acel13127-bib-0027] Jakubzick, C. V. , Randolph, G. J. , & Henson, P. M. (2017). Monocyte differentiation and antigen‐presenting functions. Nature Reviews Immunology, 17(6), 349–362. 10.1038/nri.2017.28 28436425

[acel13127-bib-0028] Jastrzebski, K. , Hannan, K. M. , Tchoubrieva, E. B. , Hannan, R. D. , & Pearson, R. B. (2007). Coordinate regulation of ribosome biogenesis and function by the ribosomal protein S6 kinase, a key mediator of mTOR function. Growth Factors, 25(4), 209–226. 10.1080/08977190701779101 18092230

[acel13127-bib-0029] Johnson, N. D. , Wiener, H. W. , Smith, A. K. , Nishitani, S. , Absher, D. M. , Arnett, D. K. , … Conneely, K. N. (2017). Non‐linear patterns in age‐related DNA methylation may reflect CD4. Epigenetics, 12(6), 492–503. 10.1080/15592294.2017.1314419 28387568 PMC5501198

[acel13127-bib-0030] Kumar, A. , Gibbs, J. R. , Beilina, A. , Dillman, A. , Kumaran, R. , Trabzuni, D. , … Cookson, M. R. (2013). Age‐associated changes in gene expression in human brain and isolated neurons. Neurobiology of Aging, 34(4), 1199–1209. 10.1016/j.neurobiolaging.2012.10.021 23177596 PMC3545059

[acel13127-bib-0031] Landsman, L. , Bar‐On, L. , Zernecke, A. , Kim, K. W. , Krauthgamer, R. , Shagdarsuren, E. , … Jung, S. (2009). CX3CR1 is required for monocyte homeostasis and atherogenesis by promoting cell survival. Blood, 113(4), 963–972. 10.1182/blood-2008-07-170787 18971423

[acel13127-bib-0032] Langston, P. K. , Shibata, M. , & Horng, T. (2017). Metabolism supports macrophage activation. Frontiers in Immunology, 8, 61 10.3389/fimmu.2017.00061 28197151 PMC5281575

[acel13127-bib-0033] Liu, Y. , Ding, J. , Reynolds, L. M. , Lohman, K. , & Register, T. C. (2013). Methylomics of gene expression in human monocytes. Human Molecular Genetics, 22(24), 5065–5074. 10.1093/hmg/ddt356 23900078 PMC3836482

[acel13127-bib-0034] Liu, Y. , Reynolds, L. M. , Ding, J. , Hou, L. , Lohman, K. , Young, T. , … Stein, J. H. (2017). Blood monocyte transcriptome and epigenome analyses reveal loci associated with human atherosclerosis. Nature Communications, 8(1), 393 10.1038/s41467-017-00517-4 PMC5577184 28855511

[acel13127-bib-0035] Loewith, R. , & Hall, M. N. (2011). Target of rapamycin (TOR) in nutrient signaling and growth control. Genetics, 189(4), 1177–1201. 10.1534/genetics.111.133363 22174183 PMC3241408

[acel13127-bib-0036] López‐Otín, C. , Blasco, M. A. , Partridge, L. , Serrano, M. , & Kroemer, G. (2013). The hallmarks of aging. Cell, 153(6), 1194–1217. 10.1016/j.cell.2013.05.039 23746838 PMC3836174

[acel13127-bib-0037] MacInnes, A. W. (2016). The role of the ribosome in the regulation of longevity and lifespan extension. Wiley Interdisciplinary Reviews: RNA, 7(2), 198–212. 10.1002/wrna.1325 26732699

[acel13127-bib-0038] Magnuson, B. , Ekim, B. , & Fingar, D. C. (2012). Regulation and function of ribosomal protein S6 kinase (S6K) within mTOR signalling networks. Biochemical Journal, 441(1), 1–21. 10.1042/BJ20110892 22168436

[acel13127-bib-0039] Metcalf, T. U. , Wilkinson, P. A. , Cameron, M. J. , Ghneim, K. , Chiang, C. , Wertheimer, A. M. , … Haddad, E. K. (2017). Human monocyte subsets are transcriptionally and functionally altered in aging in response to pattern recognition receptor agonists. The Journal of Immunology, 199(4), 1405–1417. 10.4049/JIMMUNOL.1700148 28696254 PMC5548610

[acel13127-bib-0040] O'Neill, L. A. , Kishton, R. J. , & Rathmell, J. (2016). A guide to immunometabolism for immunologists. Nature Reviews Immunology, 16(9), 553–565. 10.1038/nri.2016.70 PMC5001910 27396447

[acel13127-bib-0041] Park, H. , & Jeoung, N. H. (2016). Inflammation increases pyruvate dehydrogenase kinase 4 (PDK4) expression via the Jun N‐Terminal Kinase (JNK) pathway in C2C12 cells. Biochemical and Biophysical Research Communications, 469(4), 1049–1054. 10.1016/j.bbrc.2015.12.113 26740179

[acel13127-bib-0042] Passlick, B. , Flieger, D. , & Ziegler‐Heitbrock, H. W. (1989). Identification and characterization of a novel monocyte subpopulation in human peripheral blood. Blood, 74(7), 2527–2534. 10.1182/blood.V74.7.2527.2527 2478233

[acel13127-bib-0043] Pence, B. D. , & Yarbro, J. R. (2018). Aging impairs mitochondrial respiratory capacity in classical monocytes. Experimental Gerontology, 108, 112–117. 10.1016/J.EXGER.2018.04.008 29655929

[acel13127-bib-0044] Peters, M. J. , Joehanes, R. , Pilling, L. C. , Schurmann, C. , Conneely, K. N. , & Powell, J. … Johnson, A. D. (2015). The transcriptional landscape of age in human peripheral blood. Nature Communications, 6, 8570 10.1038/ncomms9570 PMC4639797 26490707

[acel13127-bib-0045] Pillai, P. S. , Molony, R. D. , Martinod, K. , Dong, H. , Pang, I. K. , Tal, M. C. , … Iwasaki, A. (2016). Mx1 reveals innate pathways to antiviral resistance and lethal influenza disease. Science, 352(6284), 463–466. 10.1126/science.aaf3926 27102485 PMC5465864

[acel13127-bib-0046] Reynolds, L. M. , Ding, J. , Taylor, J. R. , Lohman, K. , Soranzo, N. , de la Fuente, A. , … Liu, Y. (2015). Transcriptomic profiles of aging in purified human immune cells. BMC Genomics, 16(1), 333 10.1186/s12864-015-1522-4 25898983 PMC4417516

[acel13127-bib-0047] Reynolds, L. M. , Taylor, J. R. , Ding, J. , Lohman, K. , Johnson, C. , Siscovick, D. , … Shea, S. (2014). Age‐related variations in the methylome associated with gene expression in human monocytes and T cells. Nature Communications, 5, 5366 10.1038/ncomms6366 PMC4280798 25404168

[acel13127-bib-0048] Rogakou, E. P. , Pilch, D. R. , Orr, A. H. , Ivanova, V. S. , & Bonner, W. M. (1998). DNA double‐stranded breaks induce histone H2AX phosphorylation on serine 139. Journal of Biological Chemistry, 273(10), 5858–5868. 10.1074/jbc.273.10.5858 9488723

[acel13127-bib-0049] Sadeghi, H. M. , Schnelle, J. F. , Thoma, J. K. , Nishanian, P. , & Fahey, J. L. (1999). Phenotypic and functional characteristics of circulating monocytes of elderly persons. Experimental Gerontology, 34(8), 959–970. 10.1016/S0531-5565(99)00065-0 10673149

[acel13127-bib-0050] Sansoni, P. , Vescovini, R. , Fagnoni, F. , Biasini, C. , Zanni, F. , Zanlari, L. , … Passeri, M. (2008). The immune system in extreme longevity. Experimental Gerontology, 43(2), 61–65. 10.1016/j.exger.2007.06.008 17870272

[acel13127-bib-0051] Sengupta, S. , Peterson, T. R. , & Sabatini, D. M. (2010). Regulation of the mTOR complex 1 pathway by nutrients, growth factors, and stress. Molecular Cell, 40(2), 310–322. 10.1016/j.molcel.2010.09.026 20965424 PMC2993060

[acel13127-bib-0052] Shen‐Orr, S. S. , Furman, D. , Kidd, B. A. , Hadad, F. , Lovelace, P. , Huang, Y. W. , … Davis, M. M. (2016). Defective signaling in the JAK‐STAT pathway tracks with chronic inflammation and cardiovascular risk in aging humans. Cell Systems, 3(4), 374–384.e374. 10.1016/j.cels.2016.09.009 27746093 PMC5358544

[acel13127-bib-0053] Tabas, I. , & Lichtman, A. H. (2017). Monocyte‐macrophages and T cells in atherosclerosis. Immunity, 47(4), 621–634. 10.1016/j.immuni.2017.09.008 29045897 PMC5747297

[acel13127-bib-0054] Tacke, F. , Alvarez, D. , Kaplan, T. J. , Jakubzick, C. , Spanbroek, R. , Llodra, J. , … Randolph, G. J. (2007). Monocyte subsets differentially employ CCR2, CCR5, and CX3CR1 to accumulate within atherosclerotic plaques. Journal of Clinical Investigation, 117(1), 185–194. 10.1172/JCI28549 17200718 PMC1716202

[acel13127-bib-0055] Tiffany, H. L. , Lavigne, M. C. , Cui, Y. H. , Wang, J. M. , Leto, T. L. , Gao, J. L. , & Murphy, P. M. (2001). Amyloid‐beta induces chemotaxis and oxidant stress by acting at formylpeptide receptor 2, a G protein‐coupled receptor expressed in phagocytes and brain. Journal of Biological Chemistry, 276(26), 23645–23652. 10.1074/jbc.M101031200 11316806

[acel13127-bib-0056] Tserel, L. , Kolde, R. , Limbach, M. , Tretyakov, K. , Kasela, S. , Kisand, K. , … Peterson, P. (2015). Age‐related profiling of DNA methylation in CD8+T cells reveals changes in immune response and transcriptional regulator genes. Scientific Reports, 5, 13107 10.1038/srep13107 26286994 PMC4541364

[acel13127-bib-0057] Ucar, D. , Márquez, E. J. , Chung, C. H. , Marches, R. , Rossi, R. J. , Uyar, A. , … Banchereau, J. (2017). The chromatin accessibility signature of human immune aging stems from CD8. Journal of Experimental Medicine, 214(10), 3123–3144. 10.1084/jem.20170416 28904110 PMC5626401

[acel13127-bib-0058] van den Akker, E. B. , Passtoors, W. M. , Jansen, R. , van Zwet, E. W. , Goeman, J. J. , Hulsman, M. , … Beekman, M. (2014). Meta‐analysis on blood transcriptomic studies identifies consistently coexpressed protein‐protein interaction modules as robust markers of human aging. Aging Cell, 13(2), 216–225. 10.1111/acel.12160 24119000 PMC4331790

[acel13127-bib-0059] van Eijk, K. R. , de Jong, S. , Boks, M. P. , Langeveld, T. , Colas, F. , Veldink, J. H. , … Ophoff, R. A. (2012). Genetic analysis of DNA methylation and gene expression levels in whole blood of healthy human subjects. BMC Genomics, 13, 636 10.1186/1471-2164-13-636 23157493 PMC3583143

[acel13127-bib-0060] Villani, A.‐C. , Satija, R. , Reynolds, G. , Sarkizova, S. , Shekhar, K. , Fletcher, J. , Hacohen, N. (2017). Single‐cell RNA‐seq reveals new types of human blood dendritic cells, monocytes, and progenitors. Science, 356(6335), eaah4573 10.1126/science.aah4573 28428369 PMC5775029

[acel13127-bib-0061] Vukmanovic‐Stejic, M. , Chambers, E. S. , Suárez‐Fariñas, M. , Sandhu, D. , Fuentes‐Duculan, J. , Patel, N. , … Akbar, A. N. (2018). Enhancement of cutaneous immunity during aging by blocking p38 mitogen‐activated protein (MAP) kinase–induced inflammation. Journal of Allergy and Clinical Immunology, 142(3), 844–856. 10.1016/J.JACI.2017.10.032 29155150 PMC6127037

[acel13127-bib-0062] Whitson, R. H. , Tsark, W. , Huang, T. H. , & Itakura, K. (2003). Neonatal mortality and leanness in mice lacking the ARID transcription factor Mrf‐2. Biochemical and Biophysical Research Communications, 312(4), 997–1004. 10.1016/j.bbrc.2003.11.026 14651970

[acel13127-bib-0063] Wikby, A. , Nilsson, B. O. , Forsey, R. , Thompson, J. , Strindhall, J. , Löfgren, S. , … Johansson, B. (2006). The immune risk phenotype is associated with IL‐6 in the terminal decline stage: Findings from the Swedish NONA immune longitudinal study of very late life functioning. Mechanisms of Ageing and Development, 127(8), 695–704. 10.1016/j.mad.2006.04.003 16750842

[acel13127-bib-0064] Wullschleger, S. , Loewith, R. , & Hall, M. N. (2006). TOR signaling in growth and metabolism. Cell, 124(3), 471–484. 10.1016/j.cell.2006.01.016 16469695

[acel13127-bib-0065] Wynn, T. A. , & Vannella, K. M. (2016). Macrophages in tissue repair, regeneration, and fibrosis. Immunity, 44(3), 450–462. 10.1016/j.immuni.2016.02.015 26982353 PMC4794754

[acel13127-bib-0066] Yamada, K. , Saito, M. , Matsuoka, H. , & Inagaki, N. (2007). A real‐time method of imaging glucose uptake in single, living mammalian cells. Nature Protocols, 2(3), 753–762. 10.1038/nprot.2007.76 17406637

[acel13127-bib-0067] Yamakawa, T. , Whitson, R. H. , Li, S. L. , & Itakura, K. (2008). Modulator recognition factor‐2 is required for adipogenesis in mouse embryo fibroblasts and 3T3‐L1 cells. Molecular Endocrinology, 22(2), 441–453. 10.1210/me.2007-0271 17962384 PMC5419636

[acel13127-bib-0068] Yu, Z. , Zhai, G. , Singmann, P. , He, Y. , Xu, T. , Prehn, C. , … Wang‐Sattler, R. (2012). Human serum metabolic profiles are age dependent. Aging Cell, 11(6), 960–967. 10.1111/j.1474-9726.2012.00865.x 22834969 PMC3533791

[acel13127-bib-0069] Yun, J. , & Finkel, T. (2014). Mitohormesis. Cell Metabolism, 19(5), 757–766. 10.1016/j.cmet.2014.01.011 24561260 PMC4016106

[acel13127-bib-0070] Zahn, J. M. , Sonu, R. , Vogel, H. , Crane, E. , Mazan‐Mamczarz, K. , Rabkin, R. , … Kim, S. K. (2006). Transcriptional profiling of aging in human muscle reveals a common aging signature. PLoS Genetics, 2(7), e115 10.1371/journal.pgen.0020115.eor 16789832 PMC1513263

[acel13127-bib-0071] Zigmond, E. , Varol, C. , Farache, J. , Elmaliah, E. , Satpathy, A. T. , Friedlander, G. , … Jung, S. (2012). Ly6C hi monocytes in the inflamed colon give rise to proinflammatory effector cells and migratory antigen‐presenting cells. Immunity, 37(6), 1076–1090. 10.1016/j.immuni.2012.08.026 23219392

