# Supplementary material for: Monocytes present age‐related changes in phospholipid concentration and decreased energy metabolism
Source: Aging Cell. 2020 Feb 27;19(4):e13127. doi: 10.1111/acel.13127 (PMC7189998; doi:10.1111/acel.13127)
Supplement: Supplementary file 1 — FigureS1‐S6 [file ACEL-19-e13127-s001.pdf]

Figure S1

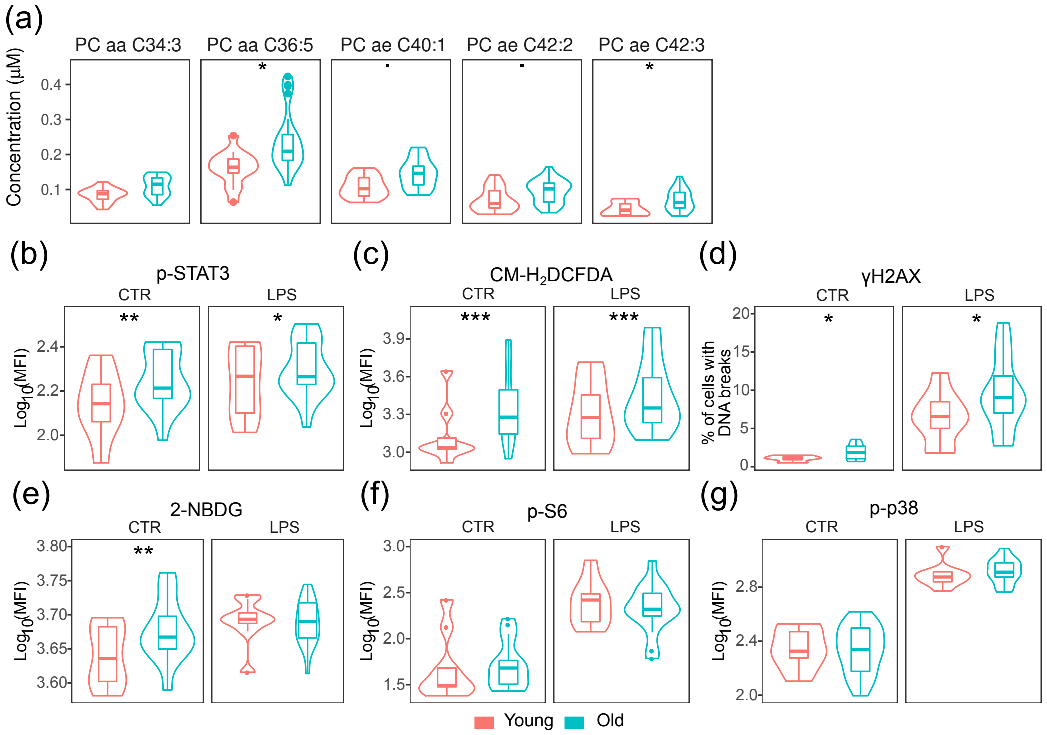

Figure S1. Lipid-lowering medication affects phosphatidylcholine concentrations in monocytes extracted from old individuals, but does not change their physiological and stress marker levels. (a) Three out of five phosphatidylcholine species that display age-related differences in concentration have weaker statistical significance when donors using lipid-lowering medication are included in the analysis. The sample size was 14 young and 23 old individuals. Monocytes were isolated from fresh samples and stored frozen until the lipid content was analyzed. Lipid-lowering medication did not alter the detection of age-related differences in the levels of (b) the inflammation marker p-STAT3, (c) oxidative stress indicator CM-H_2DCFDA,(d) the double-stranded DNA break marker γH2AX, (e) the glucose uptake indicator 2-NBDG, (f) the mTOR pathway activity marker p-S6 and (g) the general cellular stress marker p-p38 both in mock- and LPS-treated monocytes. Asterisks show the FDR-adjusted p-value ranges ·$p < 0.1$; * $p < 0.05$; ** $p < 0.01$ *** $p < 0.001$ (ANOVA).

Figure S2

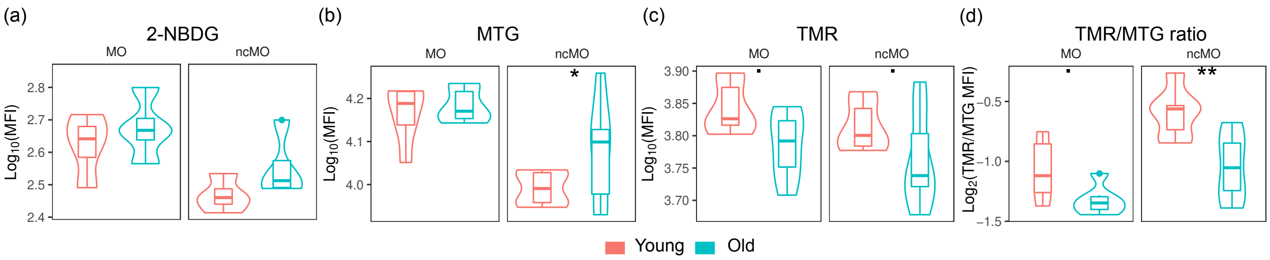

Figure S2. The age affects glucose uptake and mitochondrial function similarly in classical (MO) and non-classical (ncMO) monocytes. The cells were assesed for (a) the uptake of the glucose analog 2-NBDG, (b) mitochondrial mass, (c) mitochondrial membrane potential and (d) the ratio of mitochondrial membrane potential to mitochondrial mass. The sample sizes for each experiment were 6 young and 6 old individuals. Asterisks show the FDR-adjusted p-value ranges: $\cdot p < 0.1$; * $p < 0.05$; ** $p < 0.01$ (ANOVA).

Figure S3

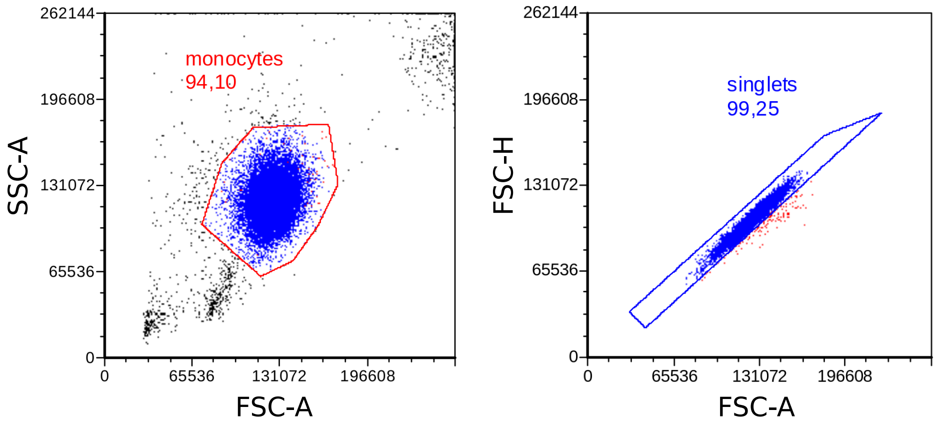

Figure S3. An illustrative example of the gating strategy to exclude dead cells and doublets. Isolated monocytes were gated according to FSC and SSC, and doublet exclusion were determined according to the area and height parameters of the FSC pulse.

Figure S4

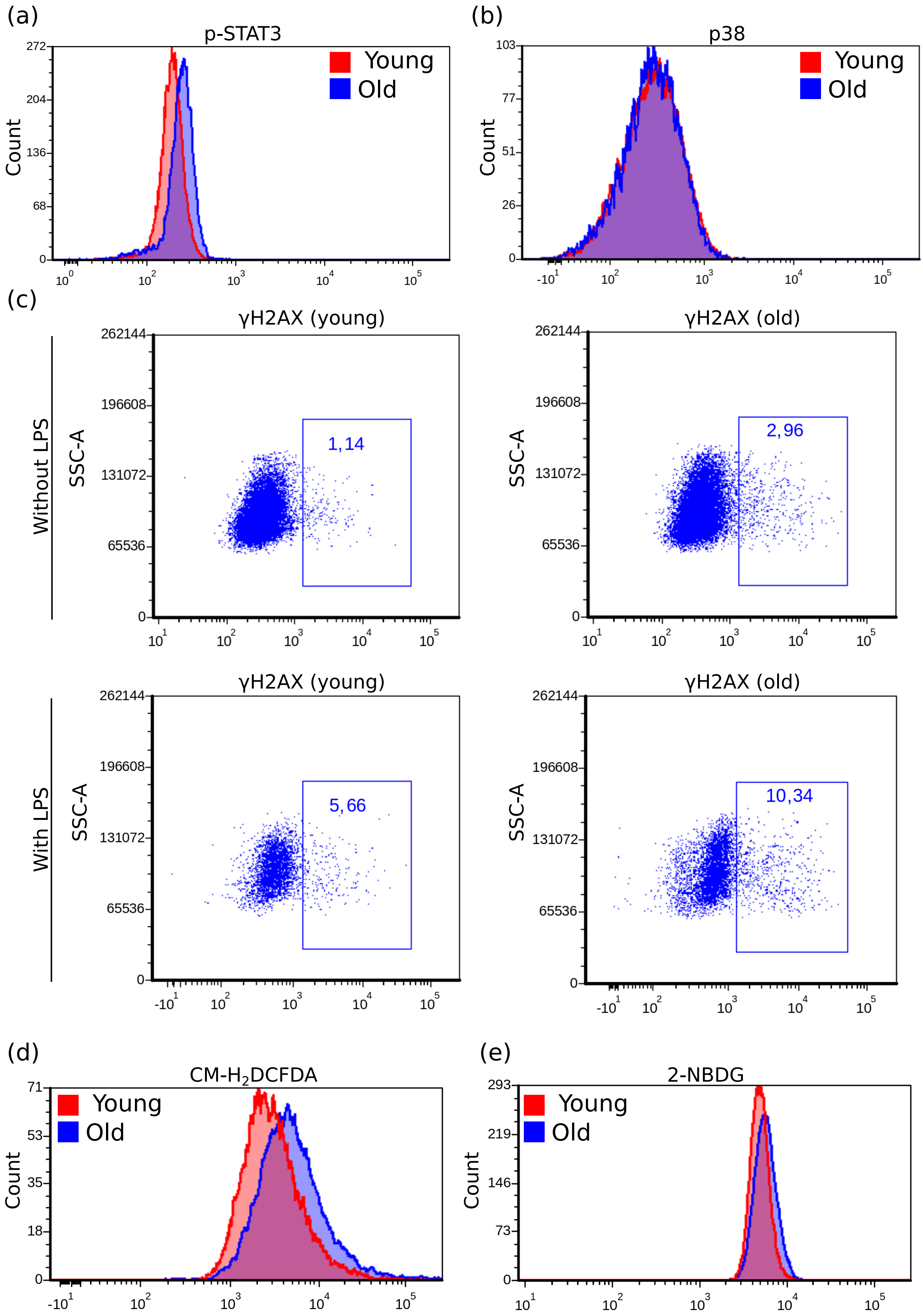
(a)
p-STAT3
Young
Old
Count
(b)
p38
Young
Old
Count
(c)
γH2AX (young)
γH2AX (old)
Without LPS
With LPS
SSC-A
1,14
2,96
5,66
10,34
(d)
CM-H2DCFDA
Young
Old
Count
(e)
2-NBDG
Young
Old
Count

Figure S4. An illustrative examples of the flow cytometry data on cellular markers of monocytes extracted from young and old individuals. a) the inflammation marker p-STAT3, (b) the general cellular stress marker p-p38, (c) he double-stranded DNA break marker γH2AX, (d) oxidative stress indicator CM-H_2DCFDA and (e) the glucose uptake indicator 2-NBDG. Please see Figure 4 in main text for the statistical analysis of the cellular markers.

Figure S5

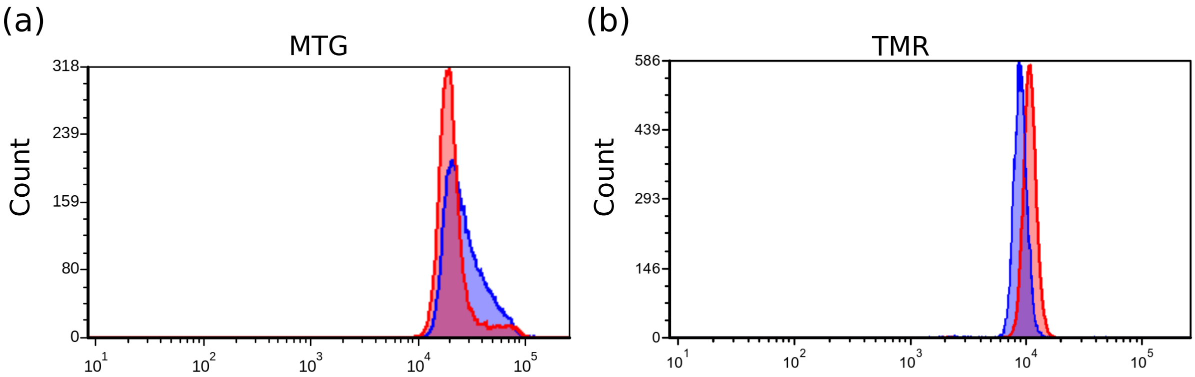

Figure S5. An illustrative examples of the flow cytometry data on mitochondrial markers of monocytes extracted from young and old individuals. a) Mitochondrial mass based on the MitoTracker Green (MTG) signal and (b) the mitochondrial membrane potential based on the tetramethylrhodamine (TMR) signal. Please see Figure 5 in main text for the statistical analysis of the mitochondrial markers.

Figure S6

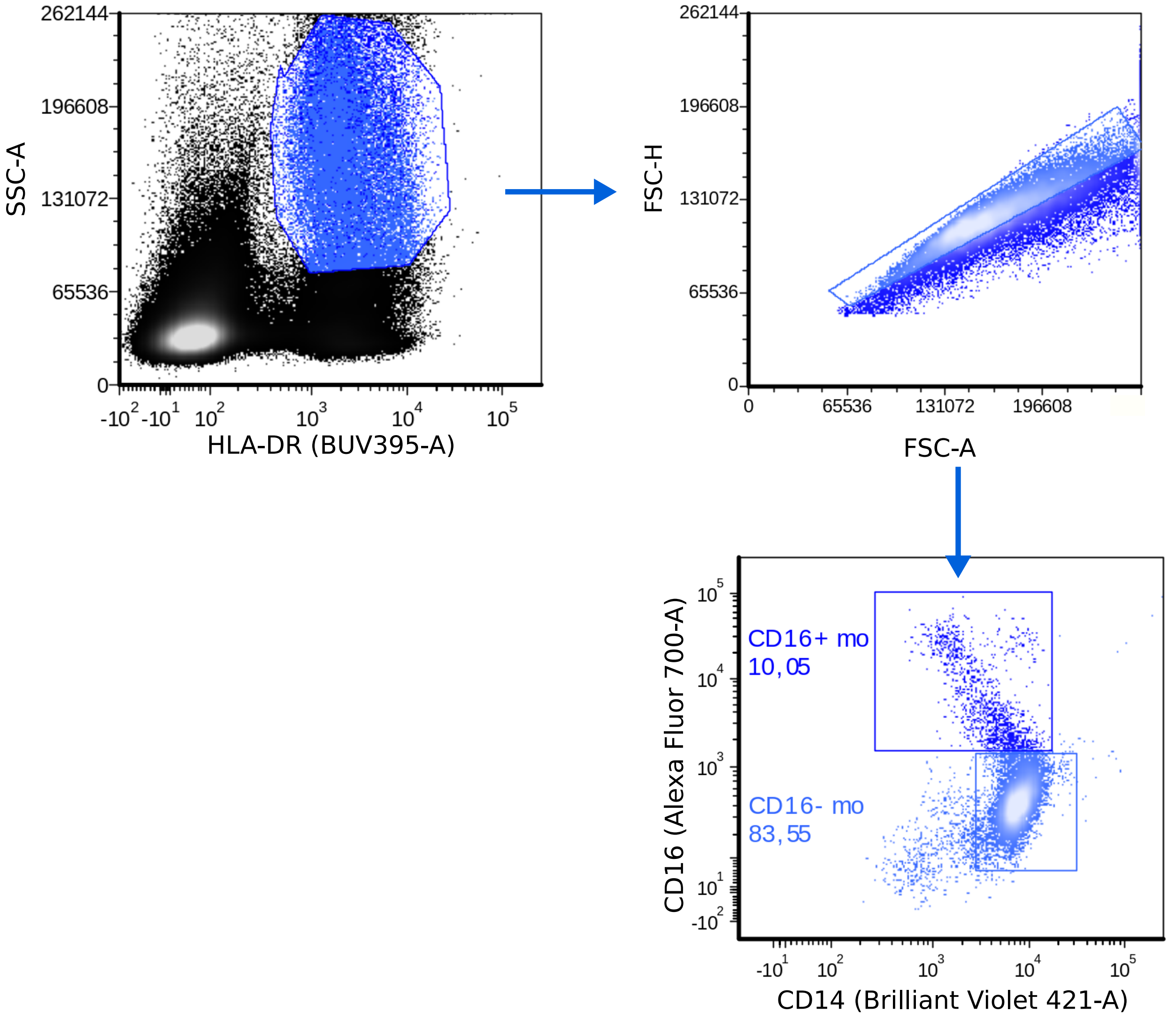

Figure S6. An illustrative example of the gating strategy to analyze conventional CD14+CD16- and non-conventional CD14+CD16+ monocytes for their metabolic and mitochondrial features. After incubation with2-NBDG, MTG or TMR, the cells were gated according to SSC and anti-HLA-DR (Brilliant UltraViolet 395) signals, and doublet exclusion were determined according to the area and height parameters of the FSC pulse. Further separation was performed on the anti-CD14 (Brillian Violet 421) and anti-CD16 (Alexa Fluor 700) antibody stainings.
