# Supplementary material for: Monocytes present age‐related changes in phospholipid concentration and decreased energy metabolism
Source: Aging Cell. 2020 Feb 27;19(4):e13127. doi: 10.1111/acel.13127 (PMC7189998; doi:10.1111/acel.13127)
Supplement: Supplementary file 10 — Supinfo [file ACEL-19-e13127-s010.docx]

Supporting Information

## Monocytes present age-related changes in phospholipid concentration and decreased energy metabolism

Mario Saare^1^, Liina Tserel^1^, Liis Haljasmägi^1^, Egon Taalberg^2^, Nadežda Peet^3^, Margus Eimre^3^, Rait Vetik^1^, Külli Kingo^4^, Kai Saks^5^, Riin Tamm^6^, Lili Milani^7^, Kai Kisand^1^* and Pärt Peterson^1^*

^1^Molecular Pathology Research Group, Institute of Biomedicine and Translational Medicine, University of Tartu, Ravila 19, 50411 Tartu, Estonia

^2^Department of Biochemistry, Institute of Biomedicine and Translational Medicine, University of Tartu, Ravila 19, 50411 Tartu, Estonia

^3^Department of Pathophysiology, Institute of Biomedicine and Translational Medicine, University of Tartu, Ravila 19, 50411 Tartu, Estonia

^4^Department of Dermatology and Venereology, Institute of Clinical Medicine, University of Tartu, Raja 31, 50417 Tartu Estonia; Clinic of Dermatology, Tartu University Hospital, Raja 31, 50417 Tartu Estonia

^5^Department of Internal Medicine, Institute of Clinical Medicine, University of Tartu, L. Puusepa 8, 50406, Tartu, Estonia

^6^Laboratory of immune analysis, United Laboratories, Tartu University Hospital, L. Puusepa 8, 50406, Tartu, Estonia

^7^Estonian Genome Center, Institute of Genomics, University of Tartu, Riia 23b/2, 51010, Tartu, Estonia

*These authors contributed equally to this work

Corresponding authors:

Mario Saare; email: mario.saare@ut.ee; telephone: +3727374186; address: Molecular Pathology Research Group, Institute of Biomedicine and Translational Medicine, University of Tartu, Ravila 19, 50411 Tartu, Estonia

Professor Kai Kisand; email: kai.kisand@ut.ee; telephone: +3727374186; address: Molecular Pathology Research Group, Institute of Biomedicine and Translational Medicine, University of Tartu, Ravila 19, 50411 Tartu, Estonia

Professor Pärt Peterson; e-mail: part.peterson@ut.ee; telephone: +3727374202; address: Molecular Pathology Research Group, Institute of Biomedicine and Translational Medicine, University of Tartu, Ravila 19, 50411 Tartu, Estonia

# Experimental Procedures

## Gene donors and ethics statement

The study was approved by the Ethics Review Committee of Human Research of the University of Tartu, Estonia according to the permissions no 206/T-4 (August 25, 2011), no 272/T-12 (August 21, 2017) and no 275/M-17 (November 20, 2017). All experiments were performed in compliance with the Helsinki Declaration. All participants were older than 18 and a written informed consent to participate in the study was obtained from each individual prior to recruitment. Participants were recruited from the Estonian Genome Center at the University of Tartu and from the Dermatology Clinic and Internal Medicine Clinic at the Tartu University Hospital. All methods were carried out in accordance with approved guidelines. Summary information about the participants is available in **Table S1**.

## Extraction and sorting of blood cells

Peripheral blood mononuclear cells (PBMC) were extracted using Ficoll-Paque (GE Healthcare, Chicago, IL, USA) gradient centrifugation. Monocytes were extracted from PBMCs by positive separation using CD14+ microbeads on a AutoMACS device according to the manufacturer’s protocol (Miltenyi Biotec, Bergisch Gladbach, Germany). The purity of extracted monocytes was determined with fluorescence conjugated antibodies against CD14 and CD3 (Miltenyi Biotec, Bergisch Gladbach, Germany) on a FACSCalibur flow cytometer (BD Biosciences, San Jose, CA, USA) (Cossarizza et al., 2019). The cells were collected and stored as pellets in a −80 °C freezer or used immediately for functional assays.

## RNA extraction and RNA-seq library preparation

RNA was extracted from the purified and frozen monocytes using the miRNeasy Mini Kit combined with recommended RNase-free DNase I treatment (both from Qiagen, Hilden, Germany). RNA was concentrated using the Heraeus vacuum centrifugation system without heating (Heraeus, Hanau, Germany). Sequencing libraries were prepared from 500 ng of RNA with RNA integrity number > 8. Sequencing libraries were generated with the TruSeq Stranded Total RNA LT Sample Prep Kit together with Ribo Zero Gold rRNA Removal Kit according to the manufacturer’s instructions (both from Illumina, San Diego, CA, USA) with the exception that 12 PCR cycles were used for the DNA enrichment step. Paired-end sequencing (2×50 bp) was performed with Illumina HiSeq 2000, which generated on average 3.75×10^7^ sequenced fragments per sample.

## RNA-seq data analysis

Prior to alignment, the reads were trimmed to remove adapter sequences and bases with a Phred score lower than 30 using Trim Galore! software (https://www.bioinformatics.babraham.ac.uk/projects/trim_galore/). Reads were mapped to the human reference genome GRCh37 (Gencode release 19) using STAR aligner version 2.5.2 with the two-pass mode (Dobin et al., 2013). Read count tables were generated with the STAR aligner option -quantMode geneCounts. Sample quality control information and clustering by principal component analysis were used to find outliers, which were removed before the statistical testing. Differential gene expression between the experimental groups was analyzed with the approach implemented in the DESeq2 software package (Love, Huber, & Anders, 2014). To account for confounding due to batch or other technical factors, five so-called surrogate variables were estimated based on the read count data with the sva software package (Leek, Johnson, Parker, Jaffe, & Storey, 2012) and incorporated into the statistical modelling step. The resulting p-values were adjusted according to the independent hypothesis weighting procedure implemented in the IHW software package (Ignatiadis, Klaus, Zaugg, & Huber, 2016). The adjusted p-value threshold was set to 0.1. The lists of up- or downregulated genes were annotated with the g:Profiler software (Reimand et al., 2016). The data that support the findings of this study are available from the corresponding author upon reasonable request.

## DNA extraction, bisulfite conversion and Illumina 450K array

Genomic DNA was isolated from the purified cell pellets using the QIAmp DNA Mini Kit according to the manufacturer’s protocol (Qiagen, Hilden, Germany). DNA was precipitated in isopropanol, washed in 70% ethanol and resuspended in 1× TE buffer. The purity and concentration of the DNA samples were measured with the NanoDrop ND-1000 spectrophotometer (Thermo Fischer Scientific, Waltham, MA, USA). An aliquot of 500 ng of genomic DNA was treated with sodium bisulfite using the EZ DNA Methylation Kit according to the manufacturer’s instructions (Zymo Research, Irvine, CA, USA). DNA methylation was assayed using the Infinium Human Methylation 450K BeadChip technology (Illumina, San Diego, CA, USA).

## DNA methylation data analysis

Raw data processing and quality control were performed with the minfi software package (Aryee et al., 2014). Samples that failed quality control or did not match the annotated sex were removed from the dataset. In addition, cross-hybridizing and polymorphic site-containing probes (listed in (Touleimat & Tost, 2012) and (Chen et al., 2013)) and probes with a detection p- value > 0.01 were removed before normalization. The filtering retained 184 samples and 358130 probes in the dataset. The normalization was performed with the stratified quantile normalization method described in (Touleimat & Tost, 2012). The differential methylation analysis was performed with the statistical approach implemented in the limma software package (Ritchie et al., 2015). Two surrogate variables which were estimated with the sva algorithm (Leek et al., 2012) to capture unwanted variability in the data were incorporated into the statistical model together with age group as the main variable of interest. The resulting p-values were adjusted by the Benjamini-Hochberg method and differential methylation was called when the adjusted p-value was below the threshold value of 0.05. The lists of hypo- and hypermethylated differentially methylated positions (DMPs) were annotated with the GREAT software (McLean et al., 2010). The data that support the findings of this study are available from the corresponding author upon reasonable request.

## Targeted metabolomics and data analysis

The AbsoluteIDQ® p180 kit (Biocrates, Innsbruck, Austria) was used to test the concentration of 188 metabolites in monocyte lysates. Samples of 5×10^5^ cells were lysed in 12.5 μl of a mixture of 10 mM phosphate buffer (PB, pH 7.1) and 96% ethanol (15:85 ratio) by performing 3 rounds of consecutive sonication, snap-freezing and heating. Sonication was done with Bioruptor (Diagenode, Seraing, Belgium) at power level H and 5×30 s ON/OFF cycles at 4 °C. The sonicated samples were snap-frozen in liquid nitrogen for 30 s, which was followed by heating at 95 °C for 30 s. The lysates were centrifuged at 16000 g for 5 min at 4 °C and the supernatant was transferred to a new tube. An aliquot of 10 μl was further processed according to the AbsoluteIDQ® p180 kit instructions provided by the manufacturer. Only metabolites passing the limit of detection in at least 75% of samples were considered for further analysis. For the statistical comparison, the concentration values were log-transformed and the difference between the age groups was analyzed with ANOVA considering the experimental batch as a confounding factor. Outlier samples were removed before the statistical analysis. Additionally, samples from older individuals who were using lipid-lowering medication were removed from the analysis, because the treatment significantly affected the results (compare **Figure 3** and **Figure S1a**).

## Phosphatidylcholine detection assay

THP-1 cells were grown in RPMI medium at 5% CO_2_ and 37 °C. Cells were treated with either DMSO serving as a control treatment or 25 µM PACOCF_3_ (Bio-Techne, Abingdon, UK), which is a phospholipase A2 inhibitor, and incubated further for 24 h. The treated cells were then washed with 1× PBS, pelleted by centrifugation and flash-frozen in liquid nitrogen. The frozen cell pellets were processed according to the Phosphatidylcholine Assay Kit protocol (Sigma-Aldrich, St. Louis, MO, USA). The signals were measured with Labsystems Multiskan MCC/340 equipment (Thermo Fischer Scientific, Waltham, MA, USA). The statistical comparison between the treatment groups was performed with the one-sided 2-sample t-test assuming equal variance.

## Sample preparation and analysis of flow cytometry data

Flow cytometric analyses were performed with mock- and LPS-treated purified monocytes. The cells were gated to exclude debris, dead cells and doublets (**Figure S3**). Inflammation and metabolic status, cellular responsiveness to stress and DNA breaks were analyzed by measuring the phosphorylation of respective key marker proteins with specific labelled antibodies: STAT3 (PE Mouse anti-Stat3 (pY705), clone 4/P-STAT3) (**Figure S4a**), S6 (PE Mouse anti-S6 (pS235/pS236), clone N7-548), p38 (Alexa Fluor® 647 Mouse anti-p38 MAPK (pT180/pY182), clone 36/p38 (pT180/pY182)) (**Figure S4b**) (all from BD Biosciences, San Jose, CA, USA) and H2A.X (anti-H2A.X phospho (Ser139) antibody, clone 2F3) (Biolegend, San Diego, CA, USA) (**Figure S4c**). The intracellular antibody stainings were performed according to the BD Phosflow protocol for human PBMCs (http://www.bdbiosciences.com/documents/Phosflow_Protocol_for_Human_PBMCs.pdf, page 4).

Oxidative stress was assessed by measuring the fluorescence of oxidized products of chloromethyl derivative of 2',7'-dichlorodihydrofluorescein diacetate (CM-H_2_DCFDA) purchased from Thermo Fischer Scientific (Waltham, MA, USA) (**Figure S4d**). The cells were incubated with 1 µM CM-H_2_DCFDA for 30 min followed by 30 min of resting time at 37 °C. Before flow cytometry, the cells were incubated with 10 ng/ml LPS or only solvent at 37 °C for another 30 min.

Glucose uptake was determined by measuring the fluorescence of the glucose analog 2-deoxy-2-((7-nitro-2,1,3-benzoxadiazol-4-yl)amino)-d-glucose (2-NBDG) (from Thermo Fischer Scientific , Waltham, MA, USA) in glucose-free medium (**Figure S4e**). The purified monocytes were first incubated with 10 ng/ml LPS or only solvent at 37 °C for 1 h. Then, 2-NBDG was added for another 30 min at a final concentration of 10 µM.

To study the amount and functionality of mitochondria, the cells were first incubated with 10 ng/ml LPS or only solvent at 37 °C for 1 h and followed by labelling with 0.1 µM MitoTracker® Green FM (MTG) or 0.1 µM tetramethylrhodamine (TMR) for 30 min at 37 °C (both reagents from Thermo Fischer Scientific, Waltham, MA, USA) (**Figure S5**).

To compare the classical monocytes to non-classical monocytes, PBMC were incubated with MTG (0.1 uM)and TMR (0.1 uM) or 2-NBDG (10 ug/ml) in glycose-free medium for 60 min at 37 C, then immediately stained with anti-CD14 Brilliant Violet 421 (Biolegend), anti-CD16 Alexa Fluor 700 (Biolegend), anti-HLA-DR Brilliant Ultraviolet 395 (BD Biosciences) (**Figure S6**).

Stained cells were analyzed using LSRFortessa flow cytometer and FACSDiva version 6 software (BD Biosciences, San Jose, CA, USA). For the statistical comparison, the mean fluorescence intensity (MFI) values were log-transformed and the difference between age groups was analyzed with ANOVA considering the experimental batch as a confounding factor. Outlier samples were removed before the statistical analysis. Additionally, samples from older individuals who were using lipid-lowering medication were removed from the analysis of pSTAT3, CM-H_2_DCFDA, γH2AX, 2-NBDG, phosphorylated S6 and p38 levels, but it did not change the conclusions after the statistical analysis (compare **Figure 4** and **Figure S1b-g**).

## Sample preparation and analysis respirometry data

The respirometric analyses were performed with the O2k oxygraph (Oroboros Instruments, Innsbruck, Austria) equipped with a Clark electrode. The measurements were collected from samples of 10^6^ purified monocytes that were kept in glucose-free RPMI 1640 medium with 5% heat inactivated FBS and fresh L-Glutamine at 37 °C in the oxygraph chambers. After observing steady-state oxygen consumption rate (basal respiration), the ATP synthase was inhibited with 2 μg/mL oligomycin (Oly), followed by uncoupling of oxidative phosphorylation by stepwise titration of carbonyl cyanide p-trifluoromethoxyphenylhydrazone (FCCP, inducing artificial proton leakage) up to an optimal concentration of 4 μM (maximal respiration). Respiration was inhibited with 2.5 μM rotenone (Rot, inhibiting complex I activity) and 2.5 μM antimycin A (AntA; inhibiting complex III). Spare respiratory capacity (SRC) was calculated as the difference between the maximal and basal respiration levels. The difference in SRC between the age groups was analyzed with ANOVA considering the experimental batch as a confounding factor.

## Quantitative PCR and data analysis

Total RNA was isolated using the TRIzol reagent according to the protocol provided by the manufacturer (Thermo Fischer Scientific, Waltham, MA, USA). From each sample, 5 μg of total RNA was used as a template for cDNA synthesis using the SuperScript III First-Strand Synthesis kit according to the manufacturer’s protocol (Thermo Fischer Scientific, Waltham, MA, USA). The gene expression levels of AIRE target genes were detected by quantitative real-time PCR (qPCR) using Maxima SYBR Green/ROX qPCR Master Mix and the ViiA 7 real-time PCR system (all from Thermo Fischer Scientific, Waltham, MA, USA). The expression of genes of interest was normalized to the housekeeping gene *HPRT1* expression and analyzed using the comparative Ct method (Livak & Schmittgen, 2001). The following primer pairs were used: *HPRT1* forward 5’ GACTTTGCTTTCCTTGGTCAGG 3’ and reverse 5’ AGTCTGGCTTATATCCAACACTTCG 3’; *PLA2G4B* forward 5’ TCCCTGGCTCTGGGTTAGAA 3’ and reverse 5’ GTCAGAGGGGGTCACTAGGT 3’; *ALOX15B* forward 5’ AGGGCAGTTTGACTCCTGTG 3’ and reverse 5’ TCAGCAACCAGAGAGCAAGG 3’. For statistical analysis, the relative expression values were log-transformed and the difference between age groups was assessed with ANOVA considering the experimental batch as a confounding factor.

# References

Aryee, M. J., Jaffe, A. E., Corrada-Bravo, H., Ladd-Acosta, C., Feinberg, A. P., Hansen, K. D., & Irizarry, R. A. (2014). Minfi: a flexible and comprehensive Bioconductor package for the analysis of Infinium DNA methylation microarrays. *Bioinformatics, 30*(10), 1363-1369. doi:10.1093/bioinformatics/btu049

Chen, Y. A., Lemire, M., Choufani, S., Butcher, D. T., Grafodatskaya, D., Zanke, B. W., . . . Weksberg, R. (2013). Discovery of cross-reactive probes and polymorphic CpGs in the Illumina Infinium HumanMethylation450 microarray. *Epigenetics, 8*(2), 203-209. doi:10.4161/epi.23470

Cossarizza, A., Chang, H., Radbruch, A., Acs, A., Adam, D., Adam‐Klages, S., … Zychlinsky, A. (2019). Guidelines for the use of flow cytometry and cell sorting in immunological studies (second edition). European Journal of Immunology, 49(10), 1457–1973. doi: 10.1002/eji.201970107

Dobin, A., Davis, C. A., Schlesinger, F., Drenkow, J., Zaleski, C., Jha, S., . . . Gingeras, T. R. (2013). STAR: ultrafast universal RNA-seq aligner. *Bioinformatics, 29*(1), 15-21. doi:10.1093/bioinformatics/bts635

Ignatiadis, N., Klaus, B., Zaugg, J. B., & Huber, W. (2016). Data-driven hypothesis weighting increases detection power in genome-scale multiple testing. *Nat Methods, 13*(7), 577-580. doi:10.1038/nmeth.3885

Leek, J. T., Johnson, W. E., Parker, H. S., Jaffe, A. E., & Storey, J. D. (2012). The sva package for removing batch effects and other unwanted variation in high-throughput experiments. *Bioinformatics, 28*(6), 882-883. doi:10.1093/bioinformatics/bts034

Livak, K. J., & Schmittgen, T. D. (2001). Analysis of relative gene expression data using real-time quantitative PCR and the 2(-Delta Delta C(T)) Method. *Methods, 25*(4), 402-408. doi:10.1006/meth.2001.1262

Love, M. I., Huber, W., & Anders, S. (2014). Moderated estimation of fold change and dispersion for RNA-seq data with DESeq2. *Genome Biol, 15*(12), 550. doi:10.1186/s13059-014-0550-8

McLean, C. Y., Bristor, D., Hiller, M., Clarke, S. L., Schaar, B. T., Lowe, C. B., . . . Bejerano, G. (2010). GREAT improves functional interpretation of cis-regulatory regions. *Nat Biotechnol, 28*(5), 495-501. doi:10.1038/nbt.1630

Reimand, J., Arak, T., Adler, P., Kolberg, L., Reisberg, S., Peterson, H., & Vilo, J. (2016). g:Profiler-a web server for functional interpretation of gene lists (2016 update). *Nucleic Acids Res, 44*(W1), W83-89. doi:10.1093/nar/gkw199

Ritchie, M. E., Phipson, B., Wu, D., Hu, Y., Law, C. W., Shi, W., & Smyth, G. K. (2015). limma powers differential expression analyses for RNA-sequencing and microarray studies. *Nucleic Acids Res, 43*(7), e47. doi:10.1093/nar/gkv007

Touleimat, N., & Tost, J. (2012). Complete pipeline for Infinium(®) Human Methylation 450K BeadChip data processing using subset quantile normalization for accurate DNA methylation estimation. *Epigenomics, 4*(3), 325-341. doi:10.2217/epi.12.21
